# SNP-Based Genetic Risk Score Modeling Suggests No Increased Genetic Susceptibility of the Roma Population to Type 2 Diabetes Mellitus

**DOI:** 10.3390/genes10110942

**Published:** 2019-11-19

**Authors:** Nardos Abebe Werissa, Peter Piko, Szilvia Fiatal, Zsigmond Kosa, Janos Sandor, Roza Adany

**Affiliations:** 1 MTA−DE Public Health Research Group of the Hungarian Academy of Sciences, Public Health Research Institute, University of Debrecen, 4028 Debrecen, Hungary; nardos.abebe@sph.unideb.hu (N.A.W.); piko.peter@sph.unideb.hu (P.P.); 2 Doctorial School of Health Sciences, University of Debrecen, 4028 Debrecen, Hungary; 3 Department of Preventive Medicine, Faculty of Public Health, University of Debrecen, 4028 Debrecen, Hungary; fiatal.szilvia@sph.unideb.hu (S.F.); sandor.janos@sph.unideb.hu (J.S.); 4 WHO Collaborating Centre on Vulnerability and Health, University of Debrecen, 4028 Debrecen, Hungary; 5 Department of Health Visitor Methodology and Public Health, Faculty of Health, University of Debrecen, 4400 Nyíregyháza, Hungary; kosa.zsigmond@foh.unideb.hu

**Keywords:** type 2 diabetes, genetic risk score, Roma, targeted intervention, single nucleotide polymorphism

## Abstract

Background: In a previous survey, an elevated fasting glucose level (FG) and/or known type 2 diabetes mellitus (T2DM) were significantly more frequent in the Roma population than in the Hungarian general population. We assessed whether the distribution of 16 single nucleotide polymorphisms (SNPs) with unequivocal effects on the development of T2DM contributes to this higher prevalence. Methods: Genetic risk scores, unweighted (GRS) and weighted (wGRS), were computed and compared between the study populations. Associations between GRSs and FG levels and T2DM status were investigated in separate and combined study populations. Results: The Hungarian general population carried a greater genetic risk for the development of T2DM (GRS_General_ = 15.38 ± 2.70 vs. GRS_Roma_ = 14.80 ± 2.68, *p* < 0.001; wGRS_General_ = 1.41 ± 0.32 vs. wGRS_Roma_ = 1.36 ± 0.31, *p* < 0.001). In the combined population models, GRSs and wGRSs showed significant associations with elevated FG (*p* < 0.001) and T2DM (*p* < 0.001) after adjusting for ethnicity, age, sex, body mass index (BMI), high-density Lipoprotein Cholesterol (HDL-C), and triglyceride (TG). In these models, the effect of ethnicity was relatively strong on both outcomes (FG levels: βethnicity = 0.918, *p* < 0.001; T2DM status: OR_ethnicity_ = 2.484, *p* < 0.001). Conclusions: The higher prevalence of elevated FG and/or T2DM among Roma does not seem to be directly linked to their increased genetic load but rather to their environmental/cultural attributes. Interventions targeting T2DM prevention among Roma should focus on harmful environmental exposures related to their unhealthy lifestyle.

## 1. Introduction

In Europe, Roma represent the largest and the most vulnerable ethnic minority group, with an estimated number of 10–12 million, approximately six million of whom live in the European Union [1]. This minority group is concentrated in the Central and Eastern European countries, mainly in Bulgaria, Hungary, Slovakia, and Romania [2], and is the target population of much ethnicity-based research—but nonetheless, only a limited number of studies have examined their genetic risk for different diseases or phenotypes. A huge number of studies have demonstrated that Roma suffer from poor health [3], unhealthy living conditions [4], low life expectancy [5], severely limited access to health services [6,7], and discrimination [8], which are closely linked to low levels of education, a high rate of unemployment, and their low socio-economic status in general [9]. 

The significantly higher prevalence of prediabetes (PreDM)—defined as a fasting blood glucose level above the normal but below the diabetic threshold, i.e., between 5.6 and 6.9 mmol/L [10]—and known type 2 diabetes mellitus (T2DM) is well demonstrated in our previous study on the Roma population compared with the Hungarian general population (27.09% vs. 15.56%; *p* < 0.001) [11]. In other studies, the higher prevalence of T2DM among Roma in Slovakia, Serbia, and Hungary compared to the general population (of Caucasian origin) was also demonstrated [12,13,14,15,16,17]. Although the latest review of the published literature [18] concludes that studies on T2DM prevalence are insufficient in design, and none of them reach the necessary standards regarding representative samples and number of participants, the authors raise a possible genetic risk to T2DM among Roma known to have Asian origin by accepting the theory of the increased genetic susceptibility to T2DM in different (Japanese, Chinese, and Indian) Asian populations [18,19]. 

It is generally accepted that, in addition to lifestyle/environmental conditions, genetic factors also have a considerable effect on the development of T2DM [20,21]. The age- and gender-adjusted heritability for elevated fasting blood glucose level was estimated to be 38% [22]. 

The supposition of the predisposition of Roma people to develop diabetes is in harmony with the so-called “thrifty gene” hypothesis regarding the role that specific genes have evolved to maximize metabolic efficiency, which are advantageous in periods of food scarcity but disadvantageous during times of abundance. According to the “thrifty genes” hypothesis, if a population has been starving for a long time, they become more prone to obesity, as well as to impaired glucose and fat tolerance in times of abundance. [23,24].

In the last two decades, genome-wide association and candidate gene studies have identified hundreds of single nucleotide polymorphisms (SNPs) that play a role in the development of T2DM [25,26,27,28,29,30,31,32]. These SNPs individually have modest effects on the risk of T2DM, with odds ratios (ORs) of 1.4 or less [33]; therefore, they cannot be used as risk predictors themselves. Genetic risk score (GRS, unweighted and weighted) modeling provides an opportunity to examine the cumulative effect of genetic factors on an outcome [34], because it sums the genetic risk attributed to each locus.

Population level-based investigation of genetic risk score modeling gives an opportunity to compare the degree of genetic load between different ethnicities and can shed light on how it varies across ethnic/racial groups. At present, a limited number of studies are available to examine the genetic susceptibility of T2DM in non-European populations [35,36,37,38], and none of them were performed on the Roma population [2]. 

The aim of the present study was to estimate and compare the risk allele load in the Roma and Hungarian general populations using the GRS modeling approach based on 16 SNPs related to T2DM, and to investigate whether the higher prevalence of PreDM and T2DM among Roma is due to inheritable and/or other factors. The findings of this research—in addition to contributing a better understanding of the genetic background of T2DM in different ethnic populations—can be used for identification of the groups for interventions targeting diabetes prevention in both populations. 

## 2. Materials and Methods 

### 2.1. Study Populations

The study subjects were obtained from previous studies and included 1783 samples from the Hungarian general (General) population [39] and 1170 Roma samples (living in segregated colonies in northeast Hungary, where this minority population is concentrated) [11,39]. The sample of the General population is representative for the Hungarian adult (above 20 years) population in terms of geographic, age, and sex distribution. As part of the survey, interviewer-assisted questionnaires were used to collect data on sociodemographic factors, lifestyle, and self-assessed health status. Medical histories were recorded, and each participant went through a thorough physical examination in both populations. Venous blood samples (native and EDTA-anticoagulated) were taken for laboratory and genotype investigations. Further details of sample collection are described elsewhere [11,39].

All procedures performed in studies involving human participants were in accordance with the ethical standards of the institutional and/or national research committee and with the 1964 Helsinki declaration and its later amendments or comparable ethical standards. This study was approved by the Ethical Committee of the University of Debrecen, Medical Health Sciences Centre (reference No. 2462-2006) and by the Ethical Committee of the Hungarian Scientific Council on Health (reference Nos. NKFP/1/0003/2005; 8907-O/2011-EKU). This article does not contain any studies with animals performed by any of the authors.

### 2.2. DNA Extraction 

Following the manufacturer’s instructions, we used a MagNA Pure LC system (Roche Diagnostics, Basel, Switzerland) with a MagNA Pure LC DNA Isolation Kit–Large Volume to isolate DNA from EDTA-anticoagulated blood samples. 

### 2.3. SNP Selection

A systematic literature search was conducted using online free databases (Ensembl PubMed, and HuGE Navigator) to identify the SNPs that were strongly associated with T2DM. A SNP was selected if it was found to be consistently associated with T2DM across European and non-European populations on a statistically acceptable sample size. During the SNP selection process, previously published meta-analysis results were considered to be of high priority (reported as odds ratios). 

### 2.4. Genotyping

We identified 16 SNPs (see the list of SNPs in Table 1) that had been genotyped by the service provider (Mutation Analysis Core Facility of the Karolinska University Hospital (MAF), Sweden). Genotyping was performed on a MassARRAY platform (Sequenom Inc., San Diego, CA, USA) with iPLEX Gold chemistry. Validation, concordance analysis, and quality control were conducted by the MAF, according to their protocols.

### 2.5. Statistical Analysis 

Hardy−Weinberg equilibrium (HWE) of the genotyped SNPs was examined with a chi-square test. Linkage disequilibrium (LD) between polymorphisms was tested by Haploview software (version 4.2). Deviation of the data from the normal distribution was checked using the Shapiro−Wilk test. When it was necessary, quantitative variables such as age, high-density Lipoprotein Cholesterol (HDL-C), body mass index (BMI), triglyceride (TG), and weighted genetic risk score were transformed using a two-step approach suggested by Templeton to reduce the effects of non-normality [61].

The diagnosis of PreDM was based on FG level (from 5.6 to 6.9 mmol/L), as it is specified in the consensus definition of the International Diabetes Federation [62] for metabolic syndrome [63]. The identification of persons with T2DM was based on a fasting glucose level ≥7 mmol/L or to be under T2DM treatment. Power calculations were performed by the software package Quanto 1.2.4 [64]. 

### 2.6. Computation of GRS and wGRS Values

To examine the cumulative effect of selected SNPs, unweighted (GRS) and weighted (wGRS) genetic risk scores were computed and compared in the study populations. Individuals with any missing genotype or phenotype data were excluded from the calculation.

In the GRS, based on the number of risk alleles carried, each person was assigned a score in the GRS based on the number of risk alleles carried. Thus, “0” indicated absence of the risk allele, while risk allele homozygotes were coded as genotype “2” and, heterozygotes as genotype “1”. [65]. By using these codes, a simple count score (unweighted) was calculated as described by Equation (1), in which Gi is the number of the risk alleles for the ith SNP. This model sums up all risk alleles over all loci as a summary score, assuming that all alleles have the same effect:

(1) $$GRS=\sum_{i=1}^{I}Gi$$

In the weighted approach, instead of giving equal weight to each SNP, SNPs with larger effects contributed more to the score. Equation (2) describes the calculation of the weighted genetic risk score, where average weights (wβ_i) were derived from the risk coefficient for each allele based on the relative effect size determined previously. These average weights (wβ_i) were multiplied by 0, 1, or 2 according to the number of effect alleles carried by each person (Xi) [65,66]:

(2) $$wGRS=\sum_{i=1}^{I}w\beta \_iXi$$

The average effect size estimate for wGRS calculation was computed by meta-analyses under the random-effects model using OpenMetaAnalyst software [67].

Student’s *t*-tests were used to compare the distribution of GRSs between the study groups. Associations between genetic risk scores and fasting glucose levels (as continuous) and PreDM or T2DM status (as binary, hereafter referred to as T2DM status) were investigated by multiple regression models (adjusted by age, sex, BMI, TG, HDL-C, and ethnicity as covariates) in separate and in combined study populations, as well. All regression analyses were performed using STATA statistical software (version 12). 

## 3. Results

### 3.1. Characteristics of the Study Samples

After the exclusion of subjects with no complete geno- and phenotype data, 1008 Roma and 1394 General individuals were included in the baseline comparison (more details in Table 2). 

The statistical power for individual SNPs was between 5.03 and 12.79% (see more details in Table 3). 

**Figure 1:**
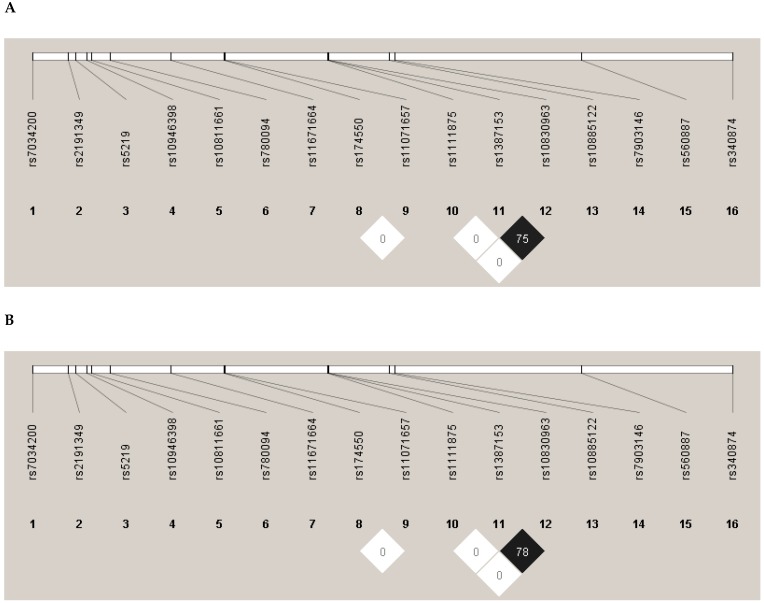
Linkage disequilibrium (LD) map of SNPs related to prediabetes/type 2 diabetes mellitus (T2DM) for the Hungarian general (**A**) and Roma (**B**) populations. Linkage analysis was performed separately in both populations, and none of the SNPs were found to be in LD. The numbers above the map show the reference SNP (rs) numbers of SNPs. Alternative coefficient of linkage disequilibrium (D′)/logarithm of odds (LOD) color scheme is used to display LD (white D′ < 1 and LOD < 2; and black D′ < 1 and LOD ≥ 2). Numbers in squares are *r*^2^ values.

In the case of the observed genotype distributions, no significant deviation from HWE was found in the study populations. None of the SNPs were in linkage disequilibrium (Figure 1).

### 3.2. Comparison of Allele Frequencies

The frequencies of SNPs’ alleles were calculated and compared between the study populations (see more details in Table 4). We found a significant difference in case of eight SNPs. Five susceptible alleles (rs7903146, rs1167664, rs340874, rs11071657, rs10946398) were more frequent in the General population and three (rs1387153, rs780094, rs10830963) among Roma.

### 3.3. Comparison of Genetic Risk Score Distributions

**Figure 2:**
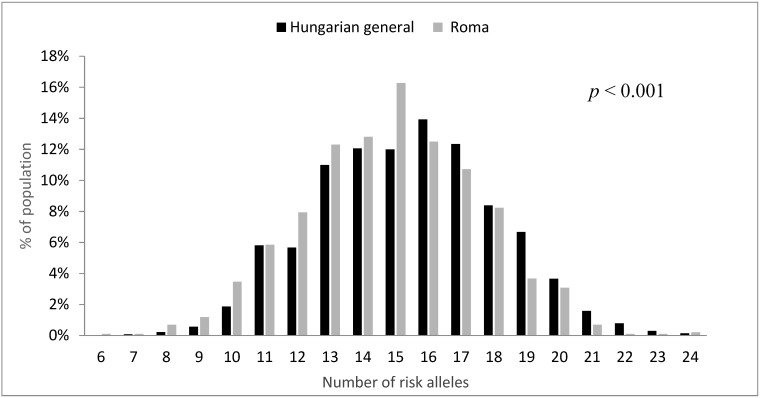
Distribution of unweighted genetic risk scores based on 16 SNPs by study population samples.

The GRS calculated for Roma individuals ranged from 6 to 24, and that for General individuals ranged from 7 to 24. The mean of the GRS was 14.8 ± 2.68 in the Roma and 15.38 ± 2.70 in the Hungarian general population samples. The distribution of the GRS in the two study groups was found to be significantly different (*p* < 0.001), being left-shifted in the Roma population relative to the General (Figure 2). 

**Figure 3:**
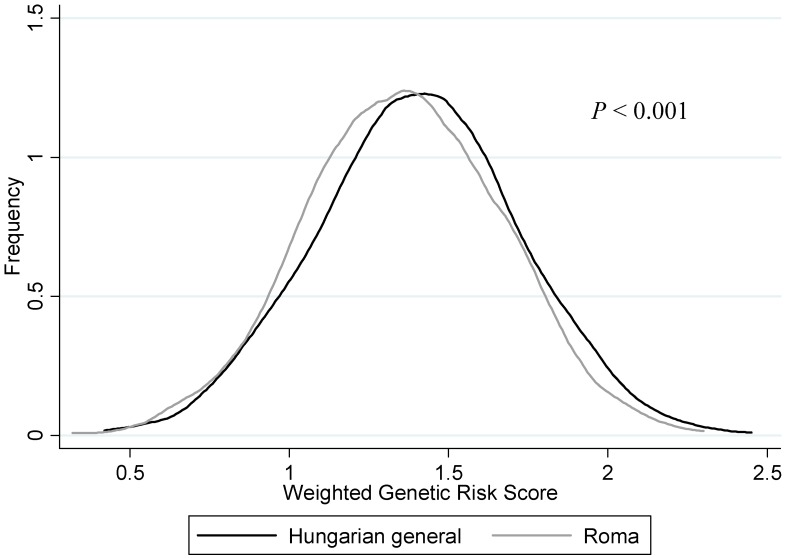
Distribution curves of weighted genetic risk scores by study population.

The average wGRS in the Roma group was 1.36 ± 0.31, while it was 1.41 ± 0.32 in the General population. The distribution of wGRSs was significantly (*p* < 0.001) different between the study populations, which is shown in Figure 3.

### 3.4. Association of Genetic Risk Scores with FG Levels and T2DM Status

Both the GRS and wGRS were analyzed for association with FG levels as a continuous variable and with T2DM status as a binary variable. The unweighted GRS was significantly associated with the two outcomes (continuous and as binary) in the adjusted model (sex, age, BMI, HDL-C, and TG levels were the covariates), both in the General (β = 0.053, *p* = 0.001; OR = 1.070, *p* = 0.027) and in the Roma (β = 0.044, *p* = 0.037; OR = 1.083, *p* = 0.010) populations (see details in Table 5). In the wGRS model, the association was significant in the General population (β = 0.489, *p* < 0.001; OR = 2.564, *p* < 0.001); however, in the case of the Roma population, a significant association was found only for T2DM status (OR = 1.932, *p* = 0.016) and not for FG levels (β = 0.300, *p* = 0.100) (see details in Table 5).

The two study populations were also jointly examined, i.e., ethnicity (General population was used as reference) was integrated into the models (Model I and II) as a covariate (besides age, sex, BMI, HDL, TG levels, and GRSs) to eliminate the effects of all ethnicity-related factors (environmental and/or cultural). In these models, the effect of GRS (Model I) and wGRS (Model II) could be examined independently from ethnicity (see detailed in Table 6). The associations between the GRS (Model I) and FG levels and T2DM status were significant (FG: β_GRS_ = 0.050, *p* < 0.001; T2DM status: OR_GRS_ = 1.075, *p* = 0.001). In the case of the weighted models (Model II), the associations were also significant (FG: β_wGRS_ = 0.425, *p* < 0.001; T2DM status: OR_wGRS_ = 2.128, *p* < 0.001).

In addition to the genetic risk score and Roma ethnicity—in harmony with previously published findings—to be a male, to be older, and to have lower HDL cholesterol and/or higher TG level have also identified as risk factors for elevated fasting glucose level and/or development of T2DM (Table 5, Table 6 and Table 7). It is important to highlight that in these multivariate models, the effect of ethnicity was relatively strong on both outcomes (FG levels: β_ethnicity_ = 0.918, *p* < 0.001; T2DM status: OR_ethnicity_ = 2.484, *p* < 0.001).

## 4. Discussion

As reported by the International Diabetes Federation, diabetes currently affects over 425 million people worldwide, and by 2045, this will rise to 625 million [62]. Type 2 diabetes accounts for at least 90% of diabetes, and its prevalence is rapidly growing, especially in low and middle income countries [45]. Studies show that not only the prevalence, but also the onset of diabetes strongly varies among ethnic groups [68,69]. On the basis of the short life expectancy of Roma and the high prevalence of metabolic syndrome among them, it was hypothesized [70] that during their prolonged migration from India to Europe, food supplies failed to meet demands, and it might have led to adaptive metabolic and genetic changes. The aim of these adaptions was the optimum utilization of scarce food supply, and so “thrifty genes” were formed in Roma. Since the time they settled down in Europe, somewhat better nutrition and reduced physical expenditure has resulted in the frequent development of metabolic syndrome with type 2 diabetes and increased cardiovascular mortality. This theory is supported by findings showing the significantly higher prevalence of metabolic syndrome [11], as well as increased cardiovascular disease (CVD) risk [71,72,73] and significantly higher mortality [74,75] among Roma. 

The present study was conducted to clarify whether the accumulation of harmful genetic factors is behind the higher prevalence of raised FG levels or T2DM status among the Roma population compared to the Hungarian general population. When the distributions of GRSs and wGRSs based on sixteen SNPs were compared between the study populations, it was shown that the General population carried greater genetic risk for developing T2DM compared with Roma. Both GRSs and wGRSs were significantly associated with FG and T2DM status in the General population, but this association was modest in the case of the Roma population. When the two populations were analyzed together (ethnicity was integrated into the model as a covariate beside age, sex, BMI, HDL-C, TG, and GRSs), the ethnicity and the GRSs had significant effects on both outcomes. By this combined analysis, the effect of ethnicity-related factors (such as lifestyle, environmental, or even unknown genetic factors) could be adjusted for. The cumulative effect of 16 SNPs involved in the GRS modeling significantly influenced the development of T2DM in the Hungarian general population, but this effect was modulated by ethnicity-related factors among Roma. 

It is generally accepted that environmental factors and unhealthy lifestyles, such as physical inactivity [76], overweight or obesity [77], and unhealthy diet [78], strongly increase the risk of developing T2DM and are linked to poor health conditions. Roma are more likely to suffer from conditions such as obesity than the general population, regardless of the country in which they live [12,79,80], but in our study, this condition did not exist. Furthermore, available reports revealed that healthy diet (relatively low intake of fats, and high consumption of fruits and vegetables) and physical activities are less common in the Roma population [81,82]. The burden of unhealthy lifestyles and cultural attributes may somewhat contribute to the high prevalence of PreDM or T2DM among Roma, but the role of still unknown genetic factors in the development of T2DM cannot be excluded.

One of the limitations of our study is that although the majority of the Roma population is accumulated in the northeast part of Hungary, our sample cannot be interpreted as a representative sample for the whole Hungarian Roma population. Assimilated Roma persons could not be excluded from the General sample population; therefore, the representative sample of the Hungarian general population included some people who are Roma. As a result of this, their inclusion might have resulted in a light underestimation of the differences between the study populations. We did not consider the exposure to epigenetic factors, gene−environmental and gene−gene interactions, as well as rare or structural variants; despite that it is well-known that all of these factors can modify genetic risk. Our analyses were adjusted only for major covariates (age, sex, BMI, HDL-C, and TG), even though there are several behavioral factors (such as physical inactivity and diet) that can obviously modify susceptibility to the studied trait. Consequently, they can account for differences in plasma FG levels between the Hungarian general and Roma populations to a certain extent. Concerning the fact that our present study was designed to define and compare the genetic risk for T2DM at the population level among the Hungarian general and Roma populations, the difference between the effect of homozygous and heterozygous gene variants on fasting glucose level and/or T2DM cannot be estimated. In our study, sixteen SNPs were considered to have considerable effects on the development of T2DM. Including a larger number of SNPs might improve the predictive ability of the genetic score model; however, it has been shown that increasing the number of SNPs in a model does not necessarily mean that the predictive ability of the model significantly increases [83,84].

Owing to the high rate of consanguinity in the Roma population [85], there might be a number of private founder mutations that are associated with increased FG level. The founder mutations identified so far are related to diseases following Mendelian inheritance. Out of these, the intron 9 +1 G>T mutation in the *SLC12A3* gene is associated with impaired glucose metabolism and significantly impaired insulin secretion in a study involving a small number of samples [86]. Nevertheless, the effects of other founder mutations—if they exist at all—cannot be excluded. 

In conclusion, this is the first study that investigated the possible genetic background of the higher prevalence of PreDM and T2DM among the Roma population. The General population carried a greater number of risk alleles relative to Roma. The cumulative effect of these genetic alterations on the development of T2DM was stronger in the General population, but in the case of Roma, the effect of inheritable factors seemed to be overwritten by ethnicity-related external factors (such as environmental and lifestyle attributes). Our findings suggest that interventions targeting T2DM prevention in the Roma population should rather focus on harmful environmental exposures related to their unhealthy lifestyle, but identifying individuals that are more susceptible to T2DM can more effectively decrease the burden related to this disease in both populations.

## Figures and Tables

**Table 1:** List of susceptible single nucleotide polymorphisms (SNPs) considered in the genetic risk score computation with their genes, effect alleles, and average effect size obtained from meta-analyses.

SNP	Gene		Effect Allele	Average Effect Size (OR)	References
rs7903146	*TCF7L2*	Transcription factor 7-like 2 (T-cell specific, HMG-box)	T	1.40	[24,40,41,42,43,44,45]
rs10811661	*CDKN2A/B*	Cyclin-Dependent Kinase Inhibitor 2A/2B	T	1.22	[24,40,46,47,48,49]
rs10946398	*CDKAL1*	CDK5 Regulatory Subunit-Associated Protein 1-Like 1	C	1.18	[46,50,51]
rs1111875	*HHEX*	Hematopoietically-Expressed Homeobox Protein	C	1.16	[52,53]
rs5219	*KCNJ11*	Potassium Voltage-Gated Channel Subfamily J Member 11	T	1.13	[54,55,56,57]
rs11671664	*GIPR*	Gastric Inhibitory Polypeptide Receptor	A	1.10	[40]
rs780094	*GCKR*	Glucokinase regulatory protein	C	1.08	[40,44,46,58]
rs1387153	*MTNR1B*	Melatonin Receptor 1B	T	1.07	[59,60]
rs340874	*PROX1*	Prospero homeobox protein 1	C	1.07	[44]
rs10830963	*MTNR1B*	Melatonin Receptor 1B	G	1.06	[40,44,58,59]
rs2191349	*DGKB–TMEM195*	Diacylglycerol Kinase Beta	T	1.06	[44]
rs174550	*FADS1*	Fatty Acid Desaturase 1	T	1.04	[44]
rs10885122	*ADRA2A*	Adrenoceptor Alpha 2A	G	1.04	[44,49]
rs11071657	*C2CD4B*	C2 Calcium-Dependent Domain-Containing 4B	A	1.03	[44]
rs7034200	*GLIS3*	GLIS Family Zinc Finger 3	A	1.03	[44]
rs560887	*G6PC2*	Glucose-6-Phosphatase Catalytic Subunit 2	T	1.03	[44]

**Table 2:** Characteristics of study populations.

Characteristics	Hungarian General Population (*N* = 1394)	Roma Population (*N* = 1008)	*p*-Value
Female	52.87	60.89	<0.001
Average age (years)	44.17 ± 12.10	40.03 ± 12.42	<0.001
BMI (kg/m^2^)	27.43 ± 5.39	26.76 ± 9.46	0.024
Fasting glucose level (mmol/L)	4.82 ± 1.67	5.28 ± 1.94	<0.001
HDL-C level (mmol/L)	1.42 ± 0.46	1.31 ± 0.41	<0.001
TG level (mmol/L)	1.64 ± 1.68	1.63 ± 1.40	0.864
Elevated fasting glucose level (>5.6 mmol/L) and/or diabetes treatment (%)	15.16	22.87	<0.001

Data are shown as the mean ± standard deviation (SD). BMI, body mass index; HDL-C, high-density Lipoprotein Cholesterol; TG, triglyceride.

**Table 3:** Statistical power of the susceptible alleles considered separately for study populations.

SNP	Gene	Effect Allele	Power for Hungarian General Population (*N* = 1394) *	Power for Roma Population (*N* = 1008) *
rs7903146	*TCF7L2*	T	12.79%	9.92%
rs10811661	*CDKN2A/B*	T	5.71%	5.45%
rs10946398	*CDKAL1*	C	5.63%	5.40%
rs1111875	*HHEX*	C	5.27%	5.19%
rs5219	*KCNJ11*	T	7.92%	7.00%
rs11671664	*GIPR*	A	5.16%	5.09%
rs780094	*GCKR*	C	5.37%	5.23%
rs1387153	*MTNR1B*	T	6.54%	6.23%
rs340874	*PROX1*	C	5.37%	5.25%
rs10830963	*MTNR1B*	C	6.77%	6.37%
rs2191349	*DGKB–TMEM195*	T	5.26%	5.19%
rs174550	*FADS1*	T	5.10%	5.07%
rs10885122	*ADRA2A*	G	5.06%	5.04%
rs11071657	*C2CD4B*	A	5.06%	5.05%
rs7034200	*GLIS3*	A	5.07%	5.05%
rs560887	*G6PC2*	T	5.03%	5.03%

* The power calculations using the software package Quanto 1.2.4 were based on the average effect sizes obtained from meta-analyses, assuming an alpha-level of 0.05 and a given sample size. In the estimation, we applied the allele frequencies for CEU (Utah Residents (CEPH) with Northern and Western Ancestry) and for GIH (Gujarati Indian from Houston, Texas) populations from the 1000 genome project, phase 3 considering that the Roma population of Europe had arrived to the Western Balkans from north India and then migrated to Europe.

**Table 4:** Comparison of susceptible allele frequencies between study populations.

SNP	Gene	Effect Allele	Allele Frequency		
			Hungarian General Population	Roma Population	*p*-Value
rs7903146	*TCF7L2*	T	29.45%	24.21%	**0.004**
rs10811661	*CDKN2A/B*	T	82.78%	85.27%	0.096
rs10946398	*CDKAL1*	C	31.36%	25.45%	**0.002**
rs1111875	*HHEX*	C	58.82%	60.62%	0.377
rs5219	*KCNJ11*	T	35.58%	32.34%	0.099
rs11671664	*GIPR*	A	11.41%	8.53%	**0.022**
rs780094	*GCKR*	C	73.64%	79.09%	**0.002**
rs1387153	*MTNR1B*	T	29.38%	35.42%	**0.002**
rs340874	*PROX1*	C	47.60%	37.45%	**<0.001**
rs10830963	*MTNR1B*	G	29.05%	33.23%	**0.029**
rs2191349	*DGKB–TMEM195*	T	57.93%	58.88%	0.636
rs174550	*FADS1*	T	70.37%	71.63%	0.504
rs10885122	*ADRA2A*	G	88.67%	89.29%	0.633
rs11071657	*C2CD4B*	A	64.06%	59.29%	**0.030**
rs7034200	*GLIS3*	A	44.62%	47.87%	0.110
rs560887	*G6PC2*	T	86.93%	86.34%	0.652

*p*-values in bold indicate at least a nominally significant difference in allele frequency between the study populations, and the higher allele frequency is shadowed in gray.

**Table 5:** Association of genetic risk scores (GRS) with fasting glucose level (**A**) and T2DM status (**B**) by study groups. The association was evaluated under adjusted regression models (sex, age, BMI, HDL-C, and TG level). OR, odds ratio; CI, confidence interval.

**A**	**Fasting Glucose Level**					
	**Hungarian General Population**			**Roma Population**		
	**β**	**95% CI**	***p*-Value**	**β**	**95% CI**	***p*-Value**
GRS	0.053	0.023–0.082	0.001	0.044	0.003−0.085	0.037
Sex (male as reference)	−0.432	−0.602 to −0.262	<0.001	−0.033	−0.264 to 0.200	0.775
Age	0.042	0.351–0.050	<0.001	0.022	0.013–0.031	<0.001
BMI	0.028	0.011–0.046	0.001	0.048	0.035–0.061	<0.001
HDL-C	−0.313	−0.532 to −0.929	0.005	−0.601	−0.899 to −0.303	<0.001
TG	0.021	−0.038–0.079	0.486	0.151	0.063–0.238	0.001
**B**	**T2DM Status**					
	**Hungarian General Population**			**Roma Population**		
	**OR**	**95% CI**	***p*-Value**	**OR**	**95% CI**	***p*-Value**
GRS	1.07	1.008–1.137	0.027	1.083	1.020–1.151	0.01
Sex (male as reference)	0.385	0.271–0.547	<0.001	0.701	0.505–0.973	0.034
Age	1.069	1.052–1.086	<0.001	1.03	1.016–1.045	<0.001
BMI	1.09	1.052–1.130	<0.001	1.031	1.012–1.051	0.001
HDL-C	0.897	0.571–1.410	0.638	0.628	0.406–0.970	0.036
TG	1.242	1.095–1.411	0.001	1.54	1.347–1.761	<0.001

**Table 6:** Association of weighted GRS (wGRS) with fasting glucose level (**A**) and T2DM status (**B**) by study groups. The association was evaluated under adjusted regression models (sex, age, BMI, HDL-C and TG level).

**A**	**Fasting Glucose Level**					
	**Hungarian General Population**			**Roma Population**		
	**β**	**95% CI**	***p*-Value**	**β**	**95% CI**	***p*-Value**
wGRS	0.489	0.240−0.738	<0.001	0.3	−0.062–0.663	0.104
Sex (male as reference)	−0.436	−0.605 to −0.266	<0.001	−0.032	−0.263 to 0.198	0.783
Age	0.043	0.035–0.050	<0.001	0.022	0.013–0.031	<0.001
BMI	0.029	0.013–0.046	0.001	0.048	0.035–0.061	<0.001
HDL-C	−0.312	−0.531 to −0.093	0.005	−0.596	−0.895 to −0.297	<0.001
TG	0.02	−0.038–0.079	0.501	0.152	0.064–0.240	0.001
**B**	**T2DM Status**					
	**Hungarian General Population**			**Roma Population**		
	**OR**	**95% CI**	***p*-Value**	**OR**	**95% CI**	***p*-Value**
wGRS	2.564	1.526–4.309	<0.001	1.932	1.133–3.292	0.016
Sex (male as reference)	0.384	0.270–0.547	<0.001	0.71	0.512–0.987	0.041
Age	1.07	1.053–1.087	<0.001	1.03	1.016–1.044	<0.001
BMI	1.091	1.052–1.131	<0.001	1.032	1.023–1.052	0.001
HDL-C	0.897	0.570–1.411	0.637	0.623	0.403–0.964	0.034
TG	1.249	1.099–1.418	0.001	1.539	1.346–1.760	<0.001

**Table 7:** Association of ethnicity (Hungarian general was used as reference) with fasting glucose level and T2DM status.

	**Fasting Glucose Level**					
	Model I *			Model II **		
	**β**	**95% CI**	***p*-Value**	**β**	**95% CI**	***p*-Value**
Ethnicity	0.918	0.779–1.058	<0.001	0.91	0.770–1.049	<0.001
GRSs	0.05	0.026–0.075	<0.001	0.425	0.216–0.634	<0.001
Sex (male as reference)	−0.262	−0.400 to −0.123	<0.001	−0.263	0.402 to −0.125	<0.001
Age	0.032	0.027–0.038	<0.001	0.032	0.027–0.038	<0.001
BMI	0.042	0.032–0.052	<0.001	0.042	0.033–0.052	<0.001
HDL-C	−0.387	−0.564 to −0.211	<0.001	−0.383	−0.560 to −0.206	<0.001
TG	0.061	0.116–0.110	0.015	0.061	0.012–0.110	0.015
	**T2DM Status**					
	Model I *			Model II **		
	**OR**	**95% CI**	***p*-Value**	**OR**	**95% CI**	***p*-Value**
Ethnicity	2.484	1.954–3.156	<0.001	2.472	1.945–3.141	<0.001
GRSs	1.075	1.031–1.121	0.001	2.128	1.477–3.067	<0.001
Sex (male as reference)	0.552	0.436–0.698	<0.001	0.554	0.438–0.701	<0.001
Age	1.047	1.036–1.058	<0.001	1.047	1.037–1.058	<0.001
BMI	1.053	1.035–1.070	<0.001	1.053	1.036–1.071	<0.001
HDL-C	0.808	0.597–1.094	0.169	0.809	0.597–1.095	0.17
TG	1.378	1.261–1.505	<0.001	1.381	1.264–1.509	<0.001

The association of ethnicity with fasting glucose level and T2DM status was evaluated under regression models (Model I and II) in the combined population (Hungarian general and Roma together). * Model I was adjusted for ethnicity and GRS, as well as sex, age, BMI, HDL-C, and TG level. ** Model II was adjusted for ethnicity and wGRS, as well as sex, age, BMI, HDL-C, and TG level.
